# Microscopic Investigation of the Combined Use of Antibiotics and Biosurfactants on Methicillin Resistant *Staphylococcus aureus*

**DOI:** 10.3389/fmicb.2020.01477

**Published:** 2020-07-07

**Authors:** Abulaziz Juma, Patrick Lemoine, Alistair B. J. Simpson, Jason Murray, Barry M. G. O’Hagan, Patrick J. Naughton, James G. Dooley, Ibrahim M. Banat

**Affiliations:** ^1^School of Biomedical Sciences, Ulster University, Coleraine, United Kingdom; ^2^School of Engineering, Nanotechnology and Integrated Bioengineering Centre (NIBEC), Ulster University, Newtownabbey, United Kingdom

**Keywords:** antibiotic, biosurfactant, *Staphylococcus aureus*, atomic force microscopy, scanning electron microscopy, synergy

## Abstract

One current strategy to deal with the serious issue of antibiotic resistance is to use biosurfactants, weak antimicrobials in their own right, with antibiotics in order to extend the efficacy of antibiotics. Although an adjuvant effect has been observed, the underlying mechanisms are poorly understood. To investigate the nature of the antibiotic and biosurfactant interaction, we undertook a scanning electron microscopy (SEM) and atomic force microscopy (AFM) microscopic study of the effects of the tetracycline antibiotic, combined with sophorolipid and rhamnolipid biosurfactants, on Methicillin-resistant *Staphylococcus aureus* using tetracycline concentrations below and above the minimum inhibitory concentration (MIC). Control and treated bacterial samples were prepared with an immersion technique by adsorbing the bacteria onto glass substrates grafted with the poly-cationic polymer polyethyleneimine. Bacterial surface morphology, hydrophobic and hydrophilic surface characters as well as the local bacterial cell stiffness were measured following combined antibiotic and biosurfactant treatment. The sophorolipid biosurfactant stands alone insofar as, when used with the antibiotic at sub-MIC concentration, it resulted in bacterial morphological changes, larger diameters (from 758 ± 75 to 1276 ± 220 nm, *p*-value = 10^–4^) as well as increased bacterial core stiffness (from 205 ± 46 to 396 ± 66 mN/m, *p*-value = 5 × 10^–5^). This investigation demonstrates that such combination of microscopic analysis can give useful information which could complement biological assays to understand the mechanisms of synergy between antibiotics and bioactive molecules such as biosurfactants.

## Introduction

Antimicrobial resistance (AMR) is a serious challenge (Tahseen et al., 2016), with a growing number of antibiotics becoming ineffective as, with time, bacteria naturally acquire resistance. Developing new antibiotics is costly and difficult. Several alternative antimicrobial strategies have been proposed such as use of bacteriophages (García et al., 2019), adaptation of antimicrobial peptides (Hollmann et al., 2018), use of phytochemicals (Monte et al., 2014) or addition of biosurfactants, such as rhamnolipids (RL) or sophorolipids (SL) (Desai and Banat, 1997). Biosurfactants which are naturally produced from micro-organisms, have long been recognized as a green, cheap, and widely available alternative to synthetic surfactants. They have environmental usages (Vijayakumar and Saravanan, 2015; Tahseen et al., 2016) and are used in the biomedical and agricultural industries, as recently reviewed (Naughton et al., 2019). Biosurfactants are indeed known antimicrobials (Díaz De Rienzo et al., 2015, 2016), although their efficacy against resistant pathogens is too low to be used on their own.

The joint use of biosurfactants and antibiotics is a recent development and published data on this synergetic effect is scarce. In one study (Rivardo et al., 2011), the biosurfactant V9T14 was used with a range of antibiotics on *E. coli*, giving a synergetic effect for sessile cells. In another, *S. aureus* was jointly treated with sophorolipid and tetracycline molecules at their respective sub-MIC levels and this resulted in an 80% reduction in colony forming units after 2 h exposure (Joshi-Navare and Prabhune, 2013). More recently (Lydon et al., 2017) sophorolipids were also found to have adjuvant activity to cefotaxime and kanamycin against planktonic *E. faecalis* and *P. aeruginosa*. Viability studies, which use standard biological assay techniques can measure cell death, biofilm disruption, etc. and quantify the efficacy of antimicrobial treatments, hence quantify the synergy between antimicrobials. However, they do not inform on the mechanisms of the combined action of antibiotics with biosurfactants, still not understood to date. As biosurfactants are surface-active agents which tend to adsorb easily on biological surfaces, their mode of action may involve modifications of the bacterial surface, hence its’ permeability to the biosurfactant and, consequently to the antibiotic, if used conjointly. Consequently, the combined effect of antibiotics and biosurfactants is best studied using high resolution microscopic techniques which can help visualize morphological changes in the bacterial surface.

In this report, we used scanning electron microscopy (SEM) and atomic force microscopy (AFM) to investigate the joint action of biosurfactants and antibiotics for both fixed and live cells. To the best of our knowledge, it is the first time that these two microscopic techniques have been used together to study such a system. The aim is to measure morphological changes of the bacterial surfaces as well as changes in local surface properties (stiffness, adhesion, measured by AFM), to help understand the effect of these antimicrobials on bacteria. The system of choice was methicillin-resistant *S. aureus* (MRSA), treated with tetracycline and RL and SL. MRSA is a human pathogen responsible for a wide range of clinical infections (Chopra and Roberts, 2001; Tong et al., 2015) and is identified by the World Health Organization (2017) as a priority 2 pathogen for research and development of new antibiotics. Tetracycline is a broad-spectrum antibiotic, cheap and extensively used therapeutically for both human and veterinary medicine (Chopra and Roberts, 2001). Tetracycline’s mode of action is to bind to the bacterial ribosome and inhibit bacterial protein synthesis (Chopra and Roberts, 2001). In the case of *S. aureus*, identified tetracycline resistance mechanisms include transmembrane efflux pumps preventing intracellular build up and alteration of the ribosome preventing binding of the drug (Bismuth et al., 1990; Chopra and Roberts, 2001; Costa et al., 2013). At this point nothing has been established for the combined action of antibiotics and biosurfactants. Hence, this investigation is focused on how RL, SL, and tetracycline interact with bacterial surfaces in adsorbed films in both fixed form through SEM and hydrated form through AFM in liquid. This is not a viability study, however, considering the scarcity of available data on such synergetic effects, it represents a useful method to identify possible modes of action and, hence screen-out candidate antimicrobials for further studies.

## Materials and Methods

### Preparation of Bacterial Films

The bacterial film were prepared on glass slides 15 × 20 mm^2^, cleaned with 70% ethanol and autoclaved. To optimize the adsorption of the bacteria on these substrates, the glass was first treated with polyethyleneimine (PEI); a polycationic polymer which has already proved successful in giving high coverage of *S. aureus* on glass (Formosa et al., 2012). This is based on *S. aureus* having an isoelectric point of 3.4; i.e., it has an overall negative surface charge at neutral pH (Petr and Maier, 2012; Horká et al., 2015) and, hence, should adhere uniformly to the positively charged PEI surface via electrostatic/Derjaguin-Landau-Verwey-Overbeek (DLVO) interactions (Brant and Childress, 2002). Hence, the clean glass slides were immersed in a 0.1% PEI solution according to manufacturers’ instructions [Merck (EMD Millipore)] allowed to dry overnight at room temperature and then stored until needed.

The *S. aureus* strain used was (MRSA) ATCC43300 with a 0.5 μg/ml minimum inhibitory concentration (MIC) (Kaur et al., 2012; Dong et al., 2017) to tetracycline (Sigma T3383). It was grown in Mueller-Hilton broth (Sigma-Aldrich, 70192) using the following procedure. Bacteria were recovered from −80°C freezer from frozen bead stocks. Bacteria were resuscitated in Muller Hinton broth and steaked out on Nutrient agar (Oxoid, CM001) for single colonies. *S. aureus* was confirmed by gram stain and biochemical tests. Muller Hinton broth was inoculated with a single colony of bacteria and incubated for 18 h at 37°C. The bacterial culture was normalized to 0.1 optical density (at 600 nm) according to CLSI requirements.

The PEI coated glass slides (as described above), positioned in sterilized petri dishes, were fully immersed in ∼10 ml of bacterial suspension at OD = 0.1 and left to incubate for 24 h at 37°C. Following incubation, the slides were removed, dried under nitrogen and stored in an airtight container at room temperature until needed. Throughout the manuscript, these bacterial samples which have not been treated with either biosurfactant or antbiotic are referred as control samples.

### Biosurfactant Characterization

The biosurfactants were extract of RL composed of Mono and di-rhamnolipids (Jeneil Biosurfactants JO LLC, product. num. JBR 425) and extract of SL (Ecover, product num. SL18-SF-D/E01-C-5.) containing a mixture of acidic and lactonic SL, obtained through personal arrangements with Jeneil Biosurfactants and Ecover company.

According to the product datasheet, the RL was produced from sterilized and centrifuged fermentation broth that has had all protein removed and partially decolorized. Two major rhamnolipids, RLL (R1 = C_26_ H_48_ O_9_) and RRLL (R2 = C_32_ H_58_ O_13_) are present. For the SL biosurfactant the manufacturer information stipulates that those correspond to a mixture of acidic and lactonic sophorolipids. Hence, we consider those biosurfactants to be clarified crude extracts and provide further compositional analysis in section “Antimicrobial Treatments.”

Liquid chromatography electrospray ionization mass spectrometry (LC /ESI /MS) is a highly selective and sensitive analysis technique (Boyaci et al., 2014) which can efficiently discriminate between different analogs and isoforms within a mixture (Yang et al., 2015). This technique was used to analyze the composition of the RL biosurfactant, using a Thermo Finnigan LCQ Classic electrospray ion – tap mass spectrometer, equipped with a 150 × 4, 6 mm Kinetex 5 μm F5 LC column. Analytical grade water and acetonitrile were used as mobile phase. The sample injection volume was 5 μl and the spectra were analyzed in positive mode from m/z 200–1000.

Matrix-assisted laser desorption/ionization time of flight mass spectrometry (MALDI-TOF-MS) is a soft ionization mass spectrometry technique that allows the identification of intact compounds (Smyth et al., 2010).

It has the advantage of high sensitivity, speed of analysis, wide applicability with a good tolerance toward contaminants (Karas, 1996). This was used to analyze the composition of the SL sample using a MDS Sciex 4800 MALDI TOF/TOF mass spectrometer from Applied Biosystems (United States). The SL sample was diluted to 0.1 mg/ml with methanol, further 10 μl of aliquot was mixed with 10 μl of CHCA matrix.

The biosurfactant’s surface activity and ability to form micelle were studied using a pendant drop technique (CAM 200 from KSV Instruments Ltd.). The size and shape of the drop depends on the competition between gravitational forces and interfacial tension, hence the surface tension of the liquid can be obtained by this technique. The system was calibrated with a glass sphere with diameter 3.90 ± 0.05 mm, giving a surface tension for DI water of 72 ± 1 mN/m. The biosurfactants were tested in solution of increasing concentration to avoid cross-contamination. The critical micelle concentration (CMC) is that at which the surface tension becomes independent of concentration. The measurements were done for a range of drop volumes, with at least five drop measurements per drop volume. The saving of the optical micrographs of the pendant drops and the subsequent estimation of the surface tension by the instrument software were carried out only a few seconds after each dispensation to avoid evaporation effects. The software also permitted to calculate the drop volume more accurately, as this variable is required in the calculation of the surface tension. Generally, it is found that one needs to use experimental conditions giving a Worthington number W_*o*_ > 0.1 to obtain valid measurements (Berry et al., 2015); where W_*o*_ is;

(1) $$W_{o}=\frac{V_{d}\cdot \Delta \rho \cdot g}{\pi \cdot \gamma \cdot D_{n}}$$

Where V_*d*_ is the drop volume, Δρ is the difference between the drop density and ambient density, g is the acceleration of gravity, γ is the surface tension of the liquid and D_*n*_ is the external diameter of the glass capillary tube. Hence, there is a trade-off between the condition W_0_ > 0.1 and the necessity to obtain stable drops. Obviously, as the biosurfactant concentration increases, the surface forces diminish and the larger drops are unstable and tend to fall off. It was found, however, that testing for drop volumes of 2–8 μl, permitted to find a range of drop volumes for which the measurement was possible.

The measurements of MIC for the two biosurfactants was carried out using a protocol adapted from a published microdilution assay (Elshikh et al., 2017). The inoculum was prepared in accordance with the CSLI recommendation where the turbidity was adjusted to the desired optical density equivalent to 1 × 10^8^ CFUml^–1^ for the bacterial culture. The columns contained 0.1 ml of the biosurfactant at the stated concentrations and were then dispensed in each well and mixed with 0.1 ml of the bacterial inoculum using a multichannel pipette. After the incubation time of 24 h at 37°C the contents of the wells were plated on Mueller Hinton agar. These plates were then once again incubated overnight at 37°C and the number of bacterial colonies was recorded.

### Antimicrobial Treatments

As antimicrobials, it was found that MIC of the RL and SL biosurfactants were, respectively, 1.25 and 2% (see “Results” below). These fairly large MIC values are indicative of these biosurfactants being weak antimicrobials in their own right, as expected.

As the focus of this microscopic study is to examine how RL and SL can assist tetracycline, rather than the other way around, the biosurfactant concentrations were kept five times below their respective MIC value; 20% of the MIC, i.e., at 0.25% for RL and 0.4% for SL. Hence, in terms of their antimicrobial effect, those biosurfactant concentrations are low. In addition, sophorolipid is recognized as having low toxicity (Kumano et al., 2019) and no deleterious effect has been observed on rats up to 4 v/v% (Ikeda et al., 1986). Similarly, rhamnolipids used here are categorized as having a category IV toxicity from the supplier (practically non-toxic and not an irritant according to the environmental protection agency (EPA) guidelines).

A number of bacterial films were prepared to investigate the effect of biosurfactants and antibiotics on the bacteria. The samples are described in Table 1. For all samples, the bacterial concentration in the final suspension corresponds to an optical density of 0.1 (at 600 nm). Except for the control samples, these solutions were prepared from more concentrated precursor solutions, so that all final solutions had the concentrations shown in Table 1.

**TABLE 1:** Sample labeling and concentrations.

Sample name	Concentrations of biosurfactant and antibiotic in final solutions
Control	Not treated
Sub	0.4 μg/ml tetracycline (~80% of MIC_*T*_)
Supra	0.6 μg/ml tetracycline (~120% of MIC_*T*_)
RL	0.25% Rhamnolipid (~20% of MIC_*RL*_
SL	0.4% Sophorolipid (~20% of MIC_*SL*_)
Sub-RL	0.4 μg/ml tetracycline and 0.25% Rhamnolipid
Sub-SL	0.4 μg/ml tetracycline and 0.4% Sophorolipid
Supra-RL	0.6 μg/ml tetracycline and 0.25% Rhamnolipid
Supra-SL	0.6 μg/ml tetracycline and 0.4% Sophorolipid

*The bacterial concentration in the final solution corresponds to an optical density of 0.1 (@ 600 nm). The MIC_T_, MIC_RL_, and MIC_SL_ are the minimal inhibitory concentration for S. aureus (ATCC 43300) for, respectively, tetracycline, rhamnolipid, and sophorolipid.*

One should note that the seemingly low tetracycline concentrations used in this study (0.4 μg/ml and 0.6 μg/ml) are in line with the MIC for tetracycline (i.e., respectively, 80 and 120% of the MIC), hence, in terms of its potential effect on *S. aureus*, and by contrast to the biosurfactants, these antimicrobial concentrations are significant.

The following procedure was adopted. The *S. aureus* control was prepared directly from the bacterial culture at 0.1 OD @ 600 nm, added to the PEI-coated slide. For all treated samples, the mixing of the base bacterial culture and antimicrobial solutions were done in a falcon tube with a roller for 15 min. The *S. aureus* samples treated with sub-MIC concentration of tetracycline (“sub” samples) were prepared by adding 0.5 ml of bacterial culture at OD = 0.2–500 μl of 0.8 μg/ml of tetracycline, mixed and poured on to the PEI-coated slide. The same process was used for the “supra” samples, but with a solution of 1.2 μg/ml of tetracycline. The RL and SL samples were prepared by forming RL and SL solutions; namely, mixing 0.5 ml of bacterial culture at OD = 0.2 with 0.5 ml of 0.5% RL solution (or 0.8% for SL) and pouring this mixture onto the PEI-coated glass slides. The sub-RL and sub-SL samples were prepared by first mixing 0.5 ml of 1% RL solution (or 1.6% for SL) with 0.5 ml of 1.6 μg/ml tetracycline solution which yielded 1 ml of 0.5%RL (or 0.8% for SL) and 0.8 μg/ml of tetracycline. Then 0.5 ml of this mixture was mixed with 0.5 ml of bacterial culture at 0.2 OD, to give a 1 ml solution with OD = 0.1, 0.25% RL (or 0.4% SL) and 0.4 μg/ml tetracycline, which was poured onto the PEI-coated slides. Finally, the two samples prepared at supra-MIC tetracycline concentration (supra-RL and supra-SL) were prepared in the same manner but using an initial 2.4 μg/ml tetracycline solution, instead of 1.6 μg/ml. All of the treated samples were stored in the fridge at 4°C directly afterward.

### SEM Microscopy

HMDS drying is a standard protocol for preparing biological samples for SEM observation (Nation, 1983; Araujo et al., 2003; Hartmann et al., 2010). In this investigation we adopted a modified version of the protocol of Hartmann et al. (2010) to ensure minimal degradation of these organic samples. The PEI-coated glass slides with adsorbed bacteria were fixed by addition of 2% glutaraldehyde [Agar Scientific, United Kingdom, v/v in phosphate buffer saline (PBS)] for 1 h at 4°C. Following fixation, each sample was washed with PBS for 5 min on ice, wash steps were completed 3 times. Sample dehydration was completed using a graded methanol series (20, 50, 70, 90, 100% methanol). We did not include the osmium tetroxide treatment as it has been shown that this step is not necessary and can even be detrimental to the cell integrity (Zhang et al., 2017). Samples were placed in ascending methanol (Sigma, United Kingdom) concentrations for 10 min then transferred to 100% hexamethyldisilazane (HMDS) (Sigma, United Kingdom) for 15 min, then left to air dry. Samples were mounted on aluminum stubs and sputter coated with a mixture of gold and palladium (ratio 80/20) for 3 min to a thickness of ∼10–15 nm using a SEM coating unit E5100 (Polaron Equipment Ltd.). SEM was carried out with a field emission system (Hitachi SU5000) using a landing voltage of 2 kV and 2 kV of deceleration and an in-lens SE detector, in order to maximize surface sensitivity and avoid sample damage. Nonetheless, a few micrographs obtained at 15 kV are also presented. Overall, more than 200 micrographs were taken, although only a selection is presented here to display the most representative features of the samples.

### AFM Microscopy

All samples examined by AFM were allowed to warm up to room temperature before measurements were made. A Bruker Dimension 3100 SPM system was used. Tapping mode atomic force microscopy (TAFM) imaging of the deposited *S. aureus* films was carried out in air with high aspect ratio Si AFM tips (half-apex angle < 25°); FESPA (∼k = 3 N/m, f = 75 kHz). These levers were operated slightly below their respective resonance with high set point/free amplitude ratio (80% in order to minimize the tip-surface interactions, tip blunting and sample damage). Contact mode atomic force microscopy (CAFM) imaging and force curve measurements were carried out in PBS using a quartz liquid cell. PBS is often the medium of choice for such experiments as it is isotonic and closely matches the pH, osmolarity and ion concentration of the human body. Two different CAFM probes were used, the Bruker MLCT-BIO-DC probes (k_*nominal*_ = 0.07 N/m, *f* = 13 kHz, *R* = 20 nm) to test adhesion against a hydrophilic material (i.e., the Si_3_N_4_ tip) and functionalized Nanosensors ST-PNP-CF_3_ probes (k_*nominal*_ = 0.08 N/m, f = 17 kHz, R = 40 nm, semi-apex angle θ∼ 15°) to test adhesion against a hydrophobic material (i.e., the CF_3_-terminated tip). In all cases, the spring constant k of the probes was calibrated with the Sader technique (Sader et al., 1995). The cantilever sensitivity was calibrated with a force curve on silicon and the AFM tip geometry of the tip was monitored using a blind reconstruction algorithm (Dongmo et al., 2000) (SPIP Software) on a standard TipCheck^®^ Sample (BudgetSensors).

CAFM images were acquired at room temperature (∼20°C), at 1 Hz scanning rate with a 0.3 V deflection set point. The force curves were acquired with a relative deflection trigger of 0.2–0.3 V at a 1 Hz frequency, equivalent to a Z displacement rate and loading rates of 1 μm/s and 30 nN/s, respectively. To ensure that force curves were carried out on target (i.e., on adsorbed bacteria), the following protocol was followed. For dense *S. aureus* coverage, mainly the control sample, the Bruker point and shoot routine was used to position the force curves on the center of each bacterium. For most other samples, where the coverage was poor, a small scan size CAFM image centered on the bacteria of interest was obtained before and after the obtention of the force curves, to ensure that the drift was minimal. In this case, a 4 × 4 array of force curves were carried out with 20 nm X and Y spacing, to keep all measurements at the center of the bacteria.

The deformation of the bacterial surface was also investigated, using the approach segments of the force curves obtained in PBS and transforming the cantilever deflection δ into the surface deformation *d = Z*-δ, where *Z* = piezo distance. This was done using the ST-PNP-CF_3_ probes with a larger radius (*R* = 40 nm) and smaller adhesion forces, allowing the use of the Hertz model (Zeng et al., 2015), from 0 to 0.5 nA force, to represent the influence of the cell wall;

(2) $$F=\frac{4}{3}\cdot \frac{E}{1-\nu^{2}}\cdot R^{1/2}\cdot d^{3/2}$$

where *F* was the load, *E* and *ν* were, respectively, the Young modulus and Poisson ratio of the bacterial cell envelope, *R* the tip radius and *d* the surface deformation. Taking a mean *ν* -value of 0.4 gave a maximal error of 10%, commensurate with the experimental error. In addition, this model remained valid as long as the contact radius *a* is smaller than *R.cos*θ = 38 nm, which was achieved on all samples for *F* < 0.5 nN. From 0.5 to 1.5 nN force, the *F-d* curve was modeled with a linear stiffness model (i.e., Hooke’s law) giving the bacterial core stiffness (S). This *S*-value represents the effect of the turgor pressure; the hydrostatic pressure resulting from the bacterial cytoplasm pushing against the cell wall. A similar approach has been recently taken to analyze AFM force curves of bacteria (Mularski et al., 2015; Gaveau et al., 2017; Formosa-Dague et al., 2018). An example of these two fitting routines is shown in Figure 13A.

### Statistical Analysis

The samples were prepared in triplicates and the statistical analysis was performed using Microsoft Excel. Data in bar charts are presented as mean and standard error for at least 10 measurements. A Student *t*-test was used to compare control and treated samples. The *p*-value was calculated assuming equal variance for both and using two tails. All results discussed in term of significant differences are those for based on the significance of variances, indicated as ^∗^ for *p*-value < 0.05, ^∗∗^ for *p*-value < 0.01 and ^∗∗∗^ for *p*-value < 0.001. As regards morphological changes observed by SEM and AFM microscopy, more than 200 images were acquired, although we only present here the most representative examples. For cases of “no morphological changes” with respect to the control bacteria (untreated), the displayed images were representative of 100% of the bacteria examined (i.e., change was never detected). For cases of “morphological changes” with respect to the control bacteria (untreated), the displayed images were representative of ∼50–70% of the bacteria examined, depending on the treatment and the nature of the change.

## Results

The compositional analysis resulting from the HPLC spectra is shown in Supplementary Figures S1, S2, which contain both spectra and table of results for the two biosurfactants. The rhamnolipid sample is mainly composed of di-rhamno-dilipidic and mono-rhamno-di-lipidic congeners whereas the sophorolipid samples is constituted of a rich mixture of lactonic and acidic congeners.

The pendant drop measurements showed that the rhamnolipid reduced the surface tension of DI water to 38 ± 3 mN/m at a CMC of 180 ± 5 ppm and that the sophorolipid reduced the surface tension of the solution to 46 ± 3 mN/m at a CMC of 177 ± 5 ppm. Previous studies found CMC values in line with the present measurements of ∼100 ppm for sophorolipid (Cho et al., 2019) and 200 ppm for rhamnolipids (Wu et al., 2019). Discrepancy between CMC values could be due to differences in congener abundance or purity as crude biosurfactants tend to have higher CMC (Jahan et al., 2020). In any case, these concentrations are well below the concentration used in this investigation, hence, the surfactants used in this microscopic study are likely to be in micellar form.

The results for the MIC measurements for the two biosurfactants are shown in Supplementary Figure S3. No bacterial numbers are detected above concentration of 1.25 and 2%, respectively, for the rhamnolipid and sophorolipid biosurfactants, hencing giving MIC_*RL*_ = 1.25% and MIC_*SL*_ = 2.00%. Previous MIC measurements of rhamnolipids and sophorolipids on *S. aureus* gave MIC_*RL*_ = 0.5% and MIC_*SL*_ = 1.00% (Díaz De Rienzo et al., 2016) although this was for a sensitive strain of *S. aureus* (ATCC 9144). It should be acknowledged that MIC values are affected by the concentration, purity and storage conditions for these compounds. They are biodegradable and often can lose some activity with time and preparation and can vary from batches and under different laboratory conditions. This said, in this study the biosurfactants are kept at concentrations five times below their respective MIC, conditions at which we expect hardly any effects from the antimicrobial treatments.

**FIGURE 1:**
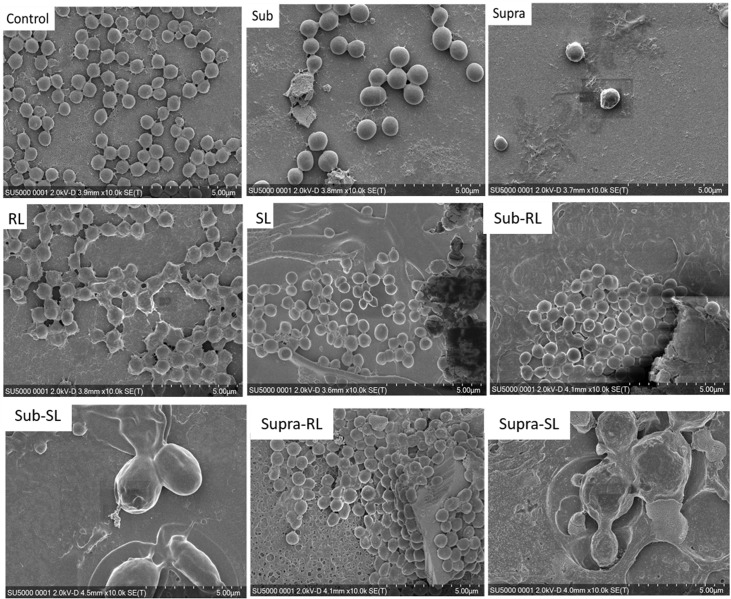
Low kV FESEM micrographs of adsorbed *S. aureus* (1010 K magnification) shown for the controlled bacteria and the various antimicrobial treatments.

**FIGURE 2:**
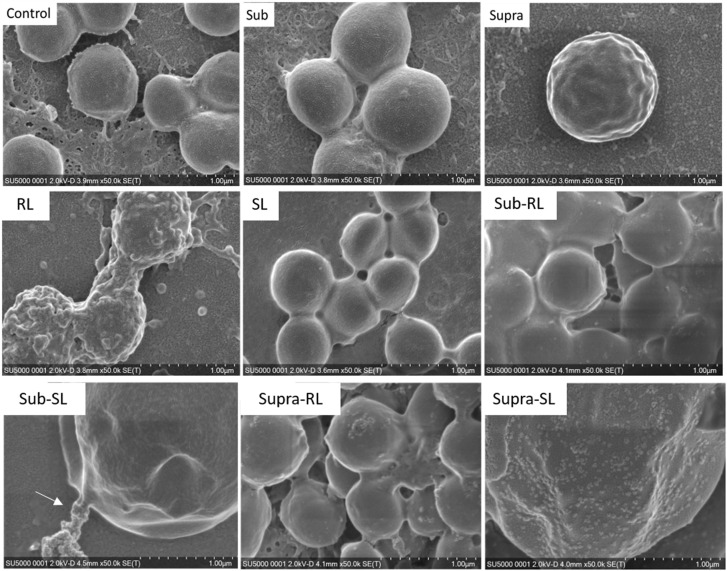
Low kV FESEM micrographs of adsorbed *S. aureus* samples (50 K magnification) shown for the controled bacteria and the various antimicrobial treatments. The Supra sample shows an enlarged bacteria with polygonal shape, the RL sample shows significant adsorption of rough deposits, in particular spherical nodules (arrows) and tthe Sub–SL sample displays some leaked material (arrow) from an enlarged bacterial cell.

**FIGURE 3:**
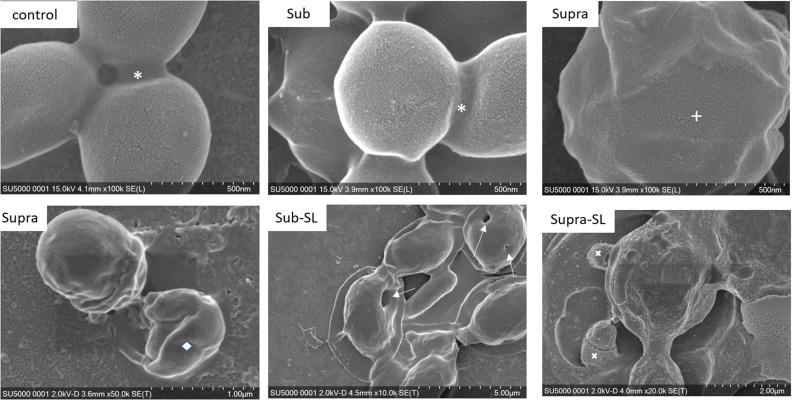
FESEM micrographs of six *S. aureus* samples (50 K magnification) of the control, sub and supra samples observed at 2 and 15 kV. For the control and Sub samples, the bacteria have their regular spherical shape with linking EPS material (*). The Supra bacteria are generally enlarged and show signs of damage such as changes to a polygonal (+) or mis-shaped (♢) morphology. Punctures (arrows) are seen on Sub-SL and leaked out cytoplasmic material (white crosses) on Supra-SL.

**FIGURE 4:**
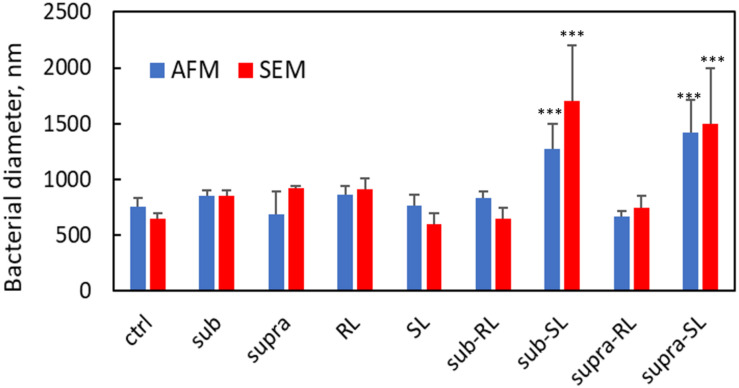
Bacterial diameters for *S. aureus* from AFM and SEM measurements. Only the Sub-SL and Supra-SL samples show statistically significant difference with the control bacteria sample.

We found that our simple preparation technique for adsorbed bacteria on PEI-treated glass allowed for a clear differentiation of the combined effects of biosurfactants and antibiotics on a multi-resistant bacterial pathogen, as observed by SEM and AFM microscopy. Figures 1, 2 show low energy SEM micrographs of the bacterial films at 10 and 50 k magnification, respectively. The micrographs of the untreated *S. aureus*, i.e., the control sample, display the expected spheroid morphologies with average diameter of ∼ 600 nm, as previously observed (Hartmann et al., 2010). They uniformly cover the surface (50–100% coverage, as expected). This is a sign of favorable interactions between the negatively charged planktonic bacteria and the positively-charged PEI-treated surface, as expected. For all other samples, the bacteria were difficult to detect. The surface coverage varied a lot, depending on the scale examined but, in all cases is much smaller than for the control bacteria (i.e., < 10%). This indicated that the tetracycline and the two biosurfactants interfered with this optimized *S. aureus*/PEI electrostatic adhesion. The bacteria treated with sub-MIC concentration of tetracycline had a similar appearance to the untreated ones, although some bacterial diameters were larger. The presence of the extracellular polymeric substance (EPS) between bacteria could be clearly seen. For those treated at supra-MIC concentration of tetracycline, the bacteria generally displayed signs of damage such as changes to a polygonal (circle) or mis-shaped (diamond) morphology, as shown on Figure 3. Some of these also had larger diameters (∼900 nm), although a bar chart of the average bacterial diameter calculated from these SEM miccrographs indicate that, overall, there are no statistically significant difference in diameter between the sample ctrl, Sub and Supra, as shown in Figure 4.

Bacteria treated with rhamnolipids were covered by rough deposits (see Figure 2). This new surface morphology is indicative of an efficient biosurfactant adsorption process, not surprising as rhamnolipids are highly tension-active. One notes spheroid nodules ∼80–100 nm diameter appearing on the bacterial surface and the surrounding PEI-glass surface. However, Figure 2 also shows that the bacteria treated with rhamnolipid have approximately the same size and diameter than the control bacteria, hence the observed morphological surface changes are not necessarily indicative of bacterial damage. Bacteria treated with sophorolipids were also similar to control bacteria with smooth surfaces and little morphological change in this case.

Once the antibiotic and biosurfactants were used together, the trends were very different. The rhamnolipid in the sub-RL and supra-RL samples did not change the morphology of the adsorbed bacterial cells, they retain a shape and diameter similar to that of the control *S. aureus*. In addition, they do not present the rough deposits seen on the RL sample, an indication that tetracycline competes with the rhamnolipid biosurfactant for adsorption on the *S. aureus* surface. On the other hand, the sophorolipid, strikingly even for sub-MIC concentration of tetracycline, gave enlarged (see Figure 4) and, sometimes, damaged and punctured bacteria, and also what appears as leaked cytoplasmic material, as seen in Figures 2, 3. Enlarged diameters and damage can also be seen for the Supra-S sample in Figures 1, 2, 4.

The bacterial samples in their hydrated state were examined by AFM microscopy. In Supplementary Figure S4 are displayed 30 μm TAFM scans of the pristine and PEI-treated glass surface, respectively, showing a homogeneous PEI film covering the glass surface. The R_*q*_ and R_*a*_ roughness values are 2.1 and 4.8 nm, respectively. A small tweezer scratch permitted the measurement of the thickness of the PEI film, ∼5.5 nm as shown from the inserted height histogram.

**FIGURE 5:**
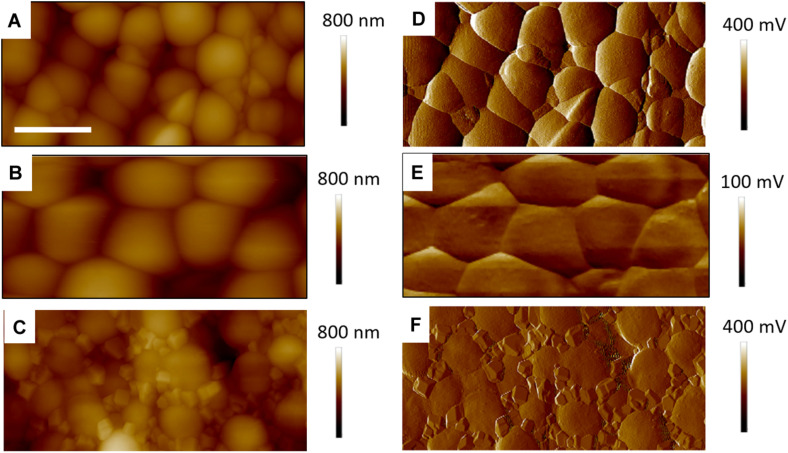
Four micrometer AFM height **(A–C)** error **(D–F)** images of control *S. aureus* for TAFM in air **(A,D)**, CAFM in PBS **(B,E)** and TAFM in air after PBS drying **(C,F)**. The error signals are, respectively, amplitude and deflection for TAFM and CAFM. The precipited salt crystals from the PBS buffer are clearly seen in **(C,F)**. For all images, micron bar = 1 μm.

In Figure 5 are gathered AFM images of the control bacteria samples, showing a dense coverage of *S. aureus* on the PEI surface. Figures 5A,D show a TAFM image obtained in air. Figures 5B,E correspond to a CAFM image obtained in PBS buffer. The morphology of the *S. aureus* bacteria was similar in both conditions, but the bacteria imaged in PBS were swollen as they were hydrated. Finally, Figures 5C,F show the same sample imaged after PBS drying and a brief DI water rinse. The bacteria have shrunk to their original size, but crystalline deposits are clearly visible between the bacteria. These are possibly NaCl crystals as it represents the main salt contained in PBS. This was not observed for any of the other *S. aureus* samples treated with antibiotic, biosurfactant or both and indicates that, for the treated samples, the PBS precipitates were either not formed or more easily rinsed. Hence, these surfaces were different from that of the control bacteria.

**FIGURE 6:**
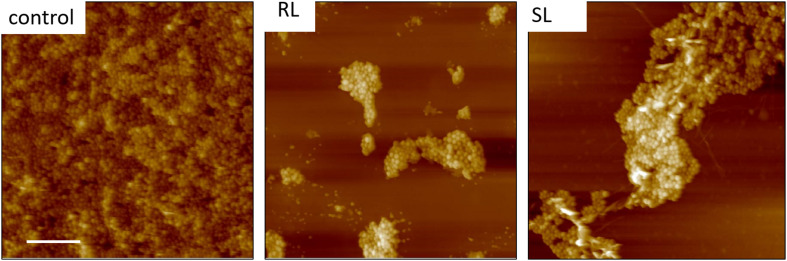
Fourty five micrometer TAFM height images in air of *S. aureus*. The bacteria treated with biosurfactants (RL and SL) show disruption in the bacterial surface coverage. Micron bar = 10 μm.

AFM microscopy on the bacterial film treated with tetracycline and/or biosurfactants proved difficult, essentially because, as observed in FESEM microscopy, the bacterial coverage was poor. Figure 6 shows TAFM images obtained in air of the control bacteria and bacteria treated with the biosurfactants alone (i.e., RL and SL samples) showing the lower coverage of the treated samples. Every deionized (DI) water rinsing and analysis in PBS resulted in a decrease of the coverage of the treated samples, making the hunting and detection of bacteria difficult and time consuming.

**FIGURE 7:**
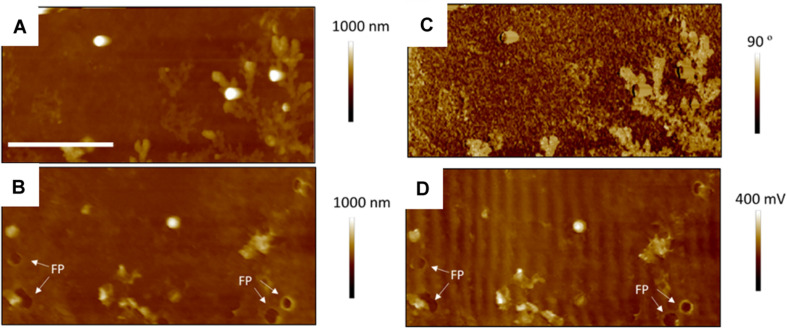
Thirty micrometer AFM images of *S. aureus* sample treated with sub-MIC concentration of tetracycline; TAFM height **(A)** and phase **(C)** image in air, CAFM height **(B)** and friction **(D)** image in PBS. Again, much fewer bacteria are seen than for the control bacteria, some have lifted off on drying, leaving footprints (FP) and dendritic precipitates are seen in the phase image. Micron bar = 10 μm.

**FIGURE 8:**
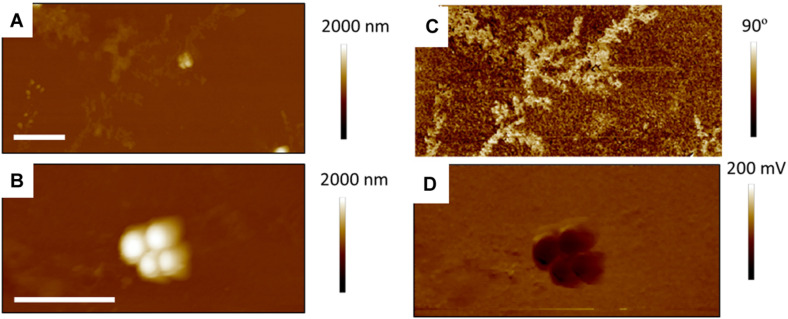
Fifteen micrometer AFM images of *S. aureus* treated with supra-MIC concentration of tetracycline; TAFM height **(A)** and phase **(C)** image in air, CAFM height **(B)** and friction **(D)** image in PBS. As for the Sub-MIC sample, there are fewer bacteria than for the control bacteria and dendritic precipitates are seen in the phase image Micron bar = 5 μm.

Figure 7 shows TAFM images in air (A,C) and CAFM images in PBS (B,D) for the *S. aureus* sample treated with sub-MIC concentration of tetracycline. Here again, the *S. aureus* coverage was poor and the bacteria were difficult to find. Branched, tree-like deposits were observed; the contrast in the phase image showing that it is a different material than the surrounding surface. Clear footprints of missing bacteria were seen for the CAFM image in PBS, indicative of the PBS buffer displacing the bacteria/PEI adhesion in this case. Cross-sections across these footprints gave a step height of 50–100 nm for the base film beneath the adsorbed bacteria, probably an adsorbed film as it is much thicker than the PEI thickness film. In Figure 8 are gathered AFM images for the *S. aureus* sample treated with supra-MIC concentration of tetracycline. Similarly, the coverage was poor and branched deposits were observed.

**FIGURE 9:**
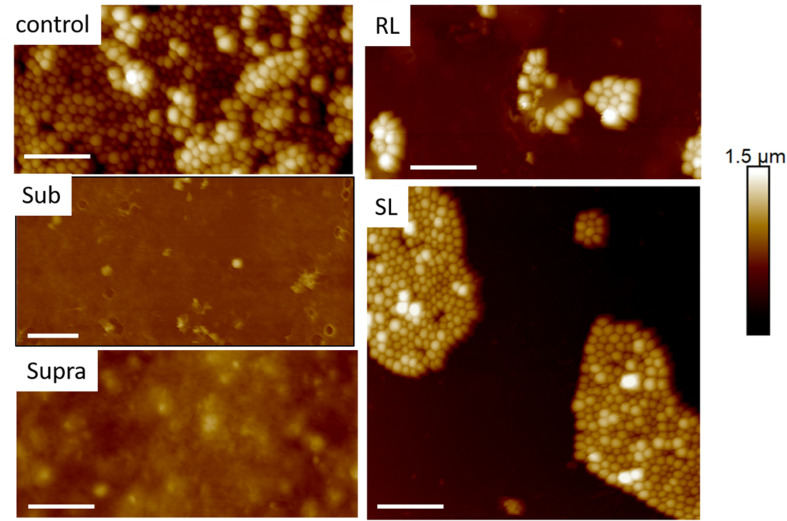
Thirty micrometer CAFM height images of *S. aureus* in PBS of control, sub, supra, RL and SL samples, same observed features than in Figures 4–8 Micron bar = 6 μm.

**FIGURE 10:**
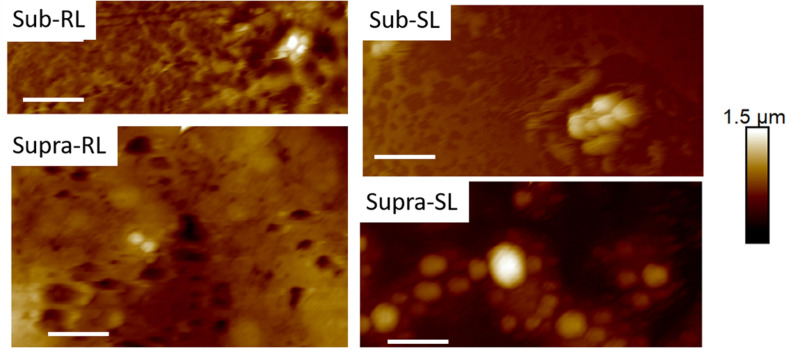
Thirty micrometer CAFM height images of *S. aureus* in PBS of Sub-RL, Sub-SL, Supra-RL and Supra-SL samples. Very few bacteria are seen on the images with some enlarged bacteria seen on the Supra-SL sample. Micron bar = 6 μm.

Figures 9, 10 show CAFM images acquired in PBS buffer for the entire batch of samples. The reduced bacterial surface coverage was observed for all treatments, as seen before on SEM micrographs. Footprints of missing bacteria are again seen on the Sub sample. One also notes that, when treated with biosurfactant alone (RL or SL), the bacteria are still clustered into islands. By contrast, the biosurfactant/tetracycline treatments resulted in very scarce coverage. In addition, the sub-S and Supra-S treatments stood out as resulting in larger adsorbed bacteria as observed in the SEM micrographs (see Figure 4).

**FIGURE 11:**
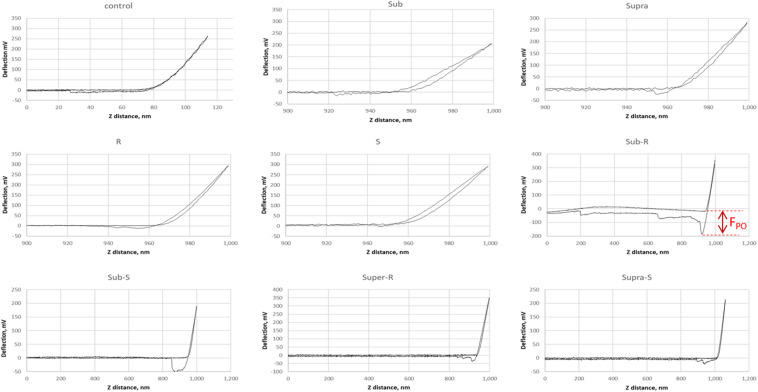
Representative AFM force curves for adsorbed *S. aureus* samples obtained in PBS buffer. The AFM cantilever had a spring constant of 0.08 N/m and was CF_3_-functionalized. The pull-off force on retraction (F_*PO*_) is measured (shown for Sub-R) and represents the tip/surface adhesion force.

**FIGURE 12:**
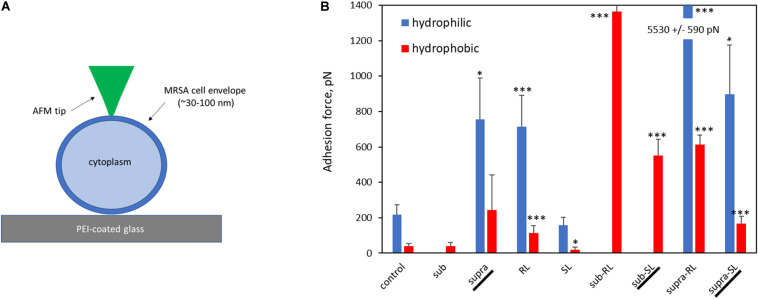
Bar chart of average AFM pull-off forces for the adsorbed *S. aureus* samples: **(A)** Scaled representation of the tip/surface contact and **(B)** Bar chart of Pull-off force (F_*PO*_) on retraction from the bacterial films, measured in PBS buffer (F_*PO*_) for both tip functionalizations. The samples underlined with a black line (Supra, Sub-SL, and Supra-SL) are those for which damaged bacteria were observed in the SEM micrographs.

Representative AFM force curves are shown in Figure 11 for the hydrophobic CF_3_-functionalized AFM tip. The averaged pull-off force values for the hydrophilic and hydrophobic AFM tips are shown in Figure 12. In this PBS environment, the pull-off forces were small, generally below 1 nN, except for the Sub-RL and Supra-RL samples. The corresponding F_*PO*_ values for hydrophilic Si_3_N_4_ AFM tips were always larger than those measured for the hydrophobic tip. Comparing the control sample to the treated ones, one notes that most *p*-values are < 0.05; most treatment brings a significant change in adhesion. Looking, more specifically at the various samples, one observes that tetracycline alone increased the adhesion force for the hydrophilic tip but no significant changes were observed for the hydrophobic tip. Rhamnolipid brought large increases of F_*PO*_ values for both tips whereas sophorolipid gave no changes. Finally, combining tetracycline with the biosurfactants gave large increases of F_*PO*_ values for both tips.

**FIGURE 13:**
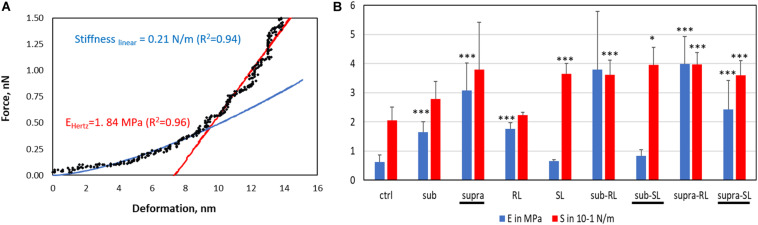
Elastic deformation of adsorbed *S. aureus* from the loading segment of the AFM force curves: **(A)** Force-deformation curve showing the Hertz contact model (0–0.5 nN) and Linear stiffness model (0.5–1.5 nN) and **(B)** Bar charts for the Young modulus (E, in MPa) and stiffness value (S in 0.1 N/m). The samples underlined with a black line (Supra, Sub-S, and Supra-S) are those for which damaged bacteria were observed in the SEM micrograph.

The deformation analysis of the bacterial surfaces is shown in Figure 13. The control bacteria has an *E*-value of 0.63 MPa whereas the treated sample generally display stiffer surfaces (up to 3.98 MPa), exept for the SL and Sub-SL treatments. The core bacterial stiffness S of the control bacteria is 0.2 N/m with, again, many treatments bringing an incease of this *S*-value. Overall these AFM measurements showed that the antibacterial treatments brought significant differences in local adhesion and stiffness of the bacterial surface but without necessarily correlating to the occurrence of morphological cell damage, as observed in the SEM mirographs.

## Discussion

A few studies have shown that there is merit in combining biosurfactants and antibiotics to address the issue of antibiotic resistance (Rivardo et al., 2011; Joshi-Navare and Prabhune, 2013; Lydon et al., 2017). The aim of the present microscopic study was to help elucidate the mechanisms of this combined action. Indeed, this comparative SEM/AFM study has permitted to obtain simultaneously high resolution images on fixed bacteria as well as rich AFM data giving topographical, mechanical and surface force information of bacteria in liquid media. The information gained is quite novel and, as will be shown in this section, allows to distinguish between biosurfactants more likely to adsorb on the external bacterial wall (i.e., rhamnolipid) and those who are probably permeating the cytoplasm (i.e., sophorolipid).

The main results exposed in the previous section have been summarized in Table 2 to outline how the various treatments have affected the control *S. aureus* bacteria.

**TABLE 2:**
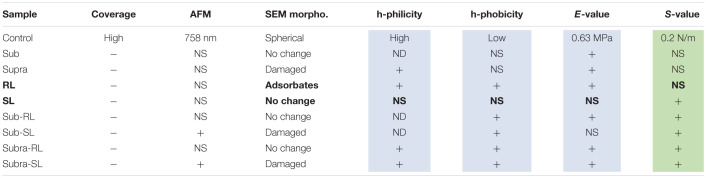
Summary of results; effects of treatments with respect to the control sample of *S. aureus.*

*ND, no data; NS, non-statistically significant difference; +, increase; -, decrease; A, adsorption; P, permeation. The blue (green) colored columns are representative of the properties of the bacterial cell envelope (cytoplasm).*

The discussion below is organized in two sections; one focused on the control bacteria, another examining the effect of the antimicrobial treatments, with references to previously published results when relevant.

### Control Bacteria

The AFM force curves showed that the control *S. aureus* surface is more hydrophilic than hydrophobic. This is consistent with the outer surface composition of gram-positive bacteria; mostly consisting of teichoic acids and cross-linked peptidoglycan chains; the amphiphilic lipoteichoic acid chains having their hydrophobic end anchored to the lipid cytoplasmic membrane (Silhavy et al., 2010; Vadillo-Rodríguez and Dutcher, 2011). The *E*-value of the control sample is 0.63 ± 0.23 MPa, calculated from the AFM force curves. It is in line with previously measured *E*-values for *S. aureus*; 0.57 MPa (Perry et al., 2009) and 0.73 MPa (Gaveau et al., 2017). Generally, as gram-positive bacteria do not have an outer membrane, they are more permeable than gram-negative species hence have a larger turgor pressure inside their cytoplasm (Vollmer et al., 2008; Osawa and Erickson, 2018). To resist this, their outer peptidoglycan envelope is thick (30–100 nm), heavily cross-linked (Silhavy et al., 2010; Saar Dover et al., 2015) and consequently stiffer than gram-negative bacteria. Indeed measured gram-negative *E*-values are generally lower, for instance *E*-values for *E. coli* are 0.25 MPa (Sokolov et al., 2006), 0.36 MPa (Chen et al., 2009), and 0.27 MPa (Oh et al., 2012). The *S*-value, 0.20 ± 0.1 N/m, representative of the turgor pressure agrees well with previous work for *S. aureus*; 0.2 N/m (Bailey et al., 2014). *S*-values for gram-negative species tend to be lower; 0.05 N/m for *P. aeruginosa* (Yao et al., 2002), 0.1 N/m for *K. pneumoniae* (Formosa-Dague et al., 2018), unless carried out in DI water which increases the turgor pressure by osmosis.

### Antimicrobial Treatments

Firstly, despite the uniform coverage of adsorbed untreated *S. aureus*, we observed a dramatic decrease of bacterial coverage for all treated bacteria; both for the dried (SEM, AFM) and hydrated (AFM) samples. The observation of bacterial footprints (Figures 7, 9) are clear signs that bacteria which had settled on the surface were later displaced. Although the PEI treatment is a generally accepted method to maximize bacterial coverage for wild strains, it is not guaranted to work as efficiently for bacteria treated by antimicrobial drugs, as the antimicrobial can interfere with the electrostatic attraction between bacteria and PEI surfaces, as experienced by others (Mularski et al., 2015). In the present case, the common hydrophilic character of tetracycline (Kavallaris et al., 1993) and PEI is likely to result in an attractive interaction. In addition, at neutral pH, tetracycline is zwitterionic (Huq et al., 2006; Zhao et al., 2011), this also can lead to a charge-dipole attraction between the PEI-surface and tetracycline. Indeed, a UV-visible spectroscopy investigation gave experimental evidence of the attractive interaction between PEI and tetracycline (Zhang et al., 2020). These effects may compete with the bacteria-PEI electrostatic attraction and lead to reduced bacterial coverage. Secondly, the biosurfactant molecules are amphiphilic, hence, can also bind easily to the charged and hydrophilic PEI surface.

Looking at the bacteria treated with supra-MIC concentration of tetracycline, its hydrophilic character increased with respect to that of the control bacteria. This could be due to adsorption of tetracycline onto the bacterial surface (i.e., both hydrophilic). In addition, the positively charged dimethyl-amino group of zwitterionic tetracycline can bond with the anionic teichoic acid chains extending though the peptidoglycan film (Silhavy et al., 2010). This increased hydrophilic character of the tetracycline-treated bacterial surfaces would increase their attraction to the PEI surface, despite the observed reduced coverage. Hence, the observation of a reduced bacterial coverage must be due to the competing and efficient tetracycline adsorption onto PEI, indeed the strong attractive PEI/tetracycline interaction has been documented before (Zhang et al., 2020). Examiming the Young modulus of the bacterial cell envelope, the *E*-value is increased by both the sub and supra treatments. There are a number of published AFM studies investigating how antimicrobials affect the Young modulus of *S. aureus*, carried out for a range of antimicrobial treatments and on different *S. aureus* strains. For some of these studies (Eaton et al., 2008; Francius et al., 2008; Chen et al., 2012), the *E*-value decreased after treatment, an expected result for these antimicrobial treatments which were all aimed at breaking or weakening the peptidoglycan bacterial envelope. In another study (Perry et al., 2009), the antimicrobials did not change the *E*-value. Increase of Young modulus upon exposure to antimicrobials has also been reported for other gram-positive bacteria (Mortensen et al., 2009; Formosa et al., 2013). In the present study, it should be noted that tetracycline’s recognized mode of action is not linked to the cell envelope but to its effect on the ribosome, hence a weakening of the cell envelope and decrease of the *E*-value is not an expected outcome, as for the case of beta lactam antibiotics, which operate by inhibiting peptidoglycan synthesis. On the other hand, if the antimicrobial molecules are trapped within this thick cross-linked peptidoglycan envelope, this would modify its mechanical properties. Indeed, when used at small concentrations, organic molecules can stiffen biopolymers. This antiplasticizing effect has been observed for the doping of carbohydrate films with sugar molecules for glycerol and starch (Chang et al., 2006) and for sorbitol and triticale proteins (Aguirre et al., 2013), and is consistent with the results observed here for the tetracycline treatments. Moreover, the AFM analysis shows that the core bacterial stiffness, the *S*-value, is unchanged by the sub and supra treatments, an indication that tetracycline brings non-detectable changes to the turgor pressure.

When the bacteria are exposed solely to the biosurfactants, we observe the following. The rhamnolipid treatment gives the largest increases in adhesive force, particularly of its hydrophilic component. As rhamnolipids are amphiphilic, singly-adsorbed rhamnolipid molecules would present their hydrophilic head to the hydrophilic *S. aureus* surface, hence result in an overall hydrophobic surface character, contrary to the observed trend. Instead, we believe that, because of the RL concentration used, the rhamnolipids are in micellar form. The obvious spherical nodules, 80–100 nm diameter, shown with arrows on Figure 2, could represent these micelles, as others have carried out dynamic light scattering experiments on the same rhamnolipids (Wu et al., 2019) and measured similar hydrodynamic diameter for these RL micelles (60–90 nm). Rhamnolipid, similar to other biosurfactants such as the recently described MSA31 lipopeptides from marine actinomycetes (Kiran et al., 2017), is also known to disrupt biofilms of *S. aureus* (Díaz De Rienzo et al., 2016). It is therefore likely to adsorb strongly on the bacteria and, hence the observed increases in F_*PO*_ values. In this case again, the adsorption onto the bacterial surface results in an increased *E*-value and no change in *S*-value. Looking now at the SL treatment, it has an insignificant effect on the F_*PO*_ and *E*-values and brings a notable increase of the *S*-value. We note that this biosurfactant is less tensio-active than rhamnolipid. This contrasting effect of the two biosurfactants, when used on their own, is outlined in Table 2; in bold for the RL and SL samples. Properties associated with the cell wall (adhesion and *E*-value) change for the RL and not for the SL sample whereas the property associated with the cytoplasm (*S*-value) changes for the SL sample and not for the RL one. This is indicative of the different effects that those two biosurfactants have on the bacteria; i.e., adsorption for RL, permeation for SL.

Examiming now the combined treatments, a striking result from the SEM micrographs is the observation of enlarged and, sometimes, damaged and punctured bacteria for the sub-SL treatment. Comparing these results to the published literature on this particular system (i.e., tetracycline and *S. aureus*), which is extremely scarce, we find no studies for joint rhamnolipid/tetracycline treatment and only one investigation for the sophorolipid/tetracycline joint treatment (Joshi-Navare and Prabhune, 2013). There are similarities between this published study and the present work; the two studies included results on bacteria treated with concentrations of antibiotic and biosurfactant, both at sub-MIC levels and, in both studies, these sub-MIC treatments resulted in bacterial damage, as observed on the SEM micrographs. There are, however, also differences between the two studies. Joshi-Navare and Prabhune use sophorolipid concentrations of 300 μg/ml for a measured MIC of 400 μg/ml, hence in relative terms, are much closer to the MIC (0.75.MIC_*SL*_) than in our study (0.2.MIC_*SL*_). In addition, the sophorolipid used in their study has a lower MIC_*SL*_, hence is a stronger antimicrobial than in our study. Finally, in the Joshi-Navare and Prabhune study, the micrographs of bacteria subjected to the combined biosurfactant/antibiotic treatment are only compared to control bacteria, hence it is not possible to identify what causes the morphological changes; the combined use or the individual treatments. By contrast, in the present study, all treatments were investigated; one can see that that the sub treatment does not modify the cell morphology. In addition, AFM data was also given to complement the information provided by the SEM micrographs. Hence, comparing the sub and sub-SL treatments (not shown on Table 2 and see Figures 12, 13) indicates that the addition of sophorolipid to tetracycline brings significant changes to both the cytoplasm (increased *S*-value) and the cell envelope (decreased *E*-value, increased F_*PO*_ value). This demonstrates that it is the combined use of both antimicrobials that is responsible for the observed changes, a result not as yet obtained on biosurfactant/antibiotic systems, to the best of our knowledge.

Looking back to the comparison with the control sample, and examining all combined treatments, Figure 12 and Table 2 clearly shows that this combination of biosurfactant and antibiotic bring significant increases in adhesive force (F_*PO*_), particularly so for the samples treated with rhamnolipid, which seemed to be again, the predominant absorbing complex. Although, these changes in surface character are not systematically accompanied by significant changes in the bacteria’s morphology, suggestive of damage to individual bacteria, they may be relevant to AMR as they could interfere with EPS release, communication between cells, and more broadly, could result in a disruption of bacterial biofilms. In addition, trapping antimicrobials within the bacterial envelope has the potential to limit their diffusion though the cytoplasm and also to block efflux pumps, which represent an important mechanism of resistance to tetracycline in *S. aureus* (Chopra and Roberts, 2001). Such a mechanism of efflux pump inhibition has been proposed to explain the synergy between essensial oils from *Chenopodium ambrosioides* leaves and tetracycline on the resistant strain *S. aureus* IS-58 (Limaverde et al., 2017). It was also noted that these combined treatments mostly bring increased *E*-value and, significantly, an increase of the *S*-value, indicative of increased turgor pressure. This can arise from a reduction of the efflux pump’s activity, as discussed above, but also from an increased permeation of the antibiotics through the cytoplasmic membrane. This can be achieved with the encapsulation of the antibiotic by a biosurfactant vesicule. This has been proposed by Joshi-Navare and Prabhune (2013) to explain the synergy between sophorolipid and cefaclor on *E. coli*. The rationale is that the hydrophobic external surface of the vesicle can interact strongly with the lipid bilayer of the outer membrane and, deliver the drug molecule to the cell interior. This process can equally operate for crossing the lipid bilayer cytoplasmic membrane of *S. aureus*. Vesicular encapsulation of antibiotics and small molecules has been demonstrated with both rhamnolipids (Pornsunthorntawee et al., 2011) and sophorolipids (Dhasaiyan et al., 2014; Haque et al., 2017). In addition, tetracycline has also been successfully encapsulated in other vesicular systems; namely phospholipid vesicles (Hasanpouri et al., 2018, Eroğlu et al., 2019) which yielded improved efficacy against *S. aureus* and *S. epidermidis*. It seems, therefore, possible, that such a process could operate for sophorolipid and tetracycline, which, therefore, is a good candidate for further studies on the synergy between tetracycline and biosurfactants for methicillin-resistant *Staphylococcus aureus*.

## Conclusion

Treating *S. aureus* films with tetracycline and rhamnolipid and sophorolipid biosurfactants dramatically reduced the bacterial coverage on PEI-treated glass surfaces. SEM micrographs and the analysis of AFM force curves suggest that, when used alone, the two biosurfactants interact differently with the bacteria, with rhamnolipid adsorbing onto the bacterial surface and sophorolipid permeating within the cytoplasm. This study also indicates that the treatment with sophorolipid and tetracycline at sub-MIC concentration resulted in swelling and morphological cell damage. This demonstrates that these two antimicrobials work co-jointly to induce cell damage. This is not observed for other antimicrobial treatments, particularly for rhamnolipid and tetracycline. AFM force curves show that overall, all antimicrobial treatments make the bacterial surface more hydrophilic. Local AFM stiffness measurements indicate that most treatments increase the stiffness of the cell wall as well as that of the bacterial core. This suggests an increase of turgor pressure. This investigation demonstrates that the joint use of sophorolipid and tetracycline is a good candidate for further studies on the synergy between tetracycline and biosurfactants. It also demonstrates that combining SEM and AFM analysis can give useful information which could complement more traditional biological assays to understand the mechanisms of synergy between antibiotics and bioactive molecules such as biosurfactants.

## Data Availability Statement

The datasets generated for this study are available on request to the corresponding author.

## Author Contributions

PL, PN, JD, and IB conceived and designed the investigation. AJ prepared the samples. BO’H and JM carried out the treatments for SEM observation. PL and AS performed the SEM and AFM experiments. PL drafted the manuscript with helpful suggestions and contributions from all. All authors contributed to the article and approved the submitted version.

## Conflict of Interest

The authors declare that the research was conducted in the absence of any commercial or financial relationships that could be construed as a potential conflict of interest.

## Supplementary Material

FIGURE S1 HPLC analysis spectrum of rhamnolipid and table of results with approximate probable molecular weights.

FIGURE S2 MALDI-TOF analysis spectrum of sophorolipid and table of results with approximate probable molecular weights.

FIGURE S3 Results for microdilution assay: The bacterial number are in CFU/ml.

FIGURE S4 30 μm TAFM height images of glass surface **(A)** and PEI-coated glass surface **(B)**. The insert shows a height histogram originating from a sample region, dashed rectangle, around a scratch.
