# Supplementary figures and images for: Microscopic Investigation of the Combined Use of Antibiotics and Biosurfactants on Methicillin Resistant Staphylococcus aureus
Source: Front Microbiol. 2020 Jul 7;11:1477. doi: 10.3389/fmicb.2020.01477 (PMC7358407; doi:10.3389/fmicb.2020.01477)

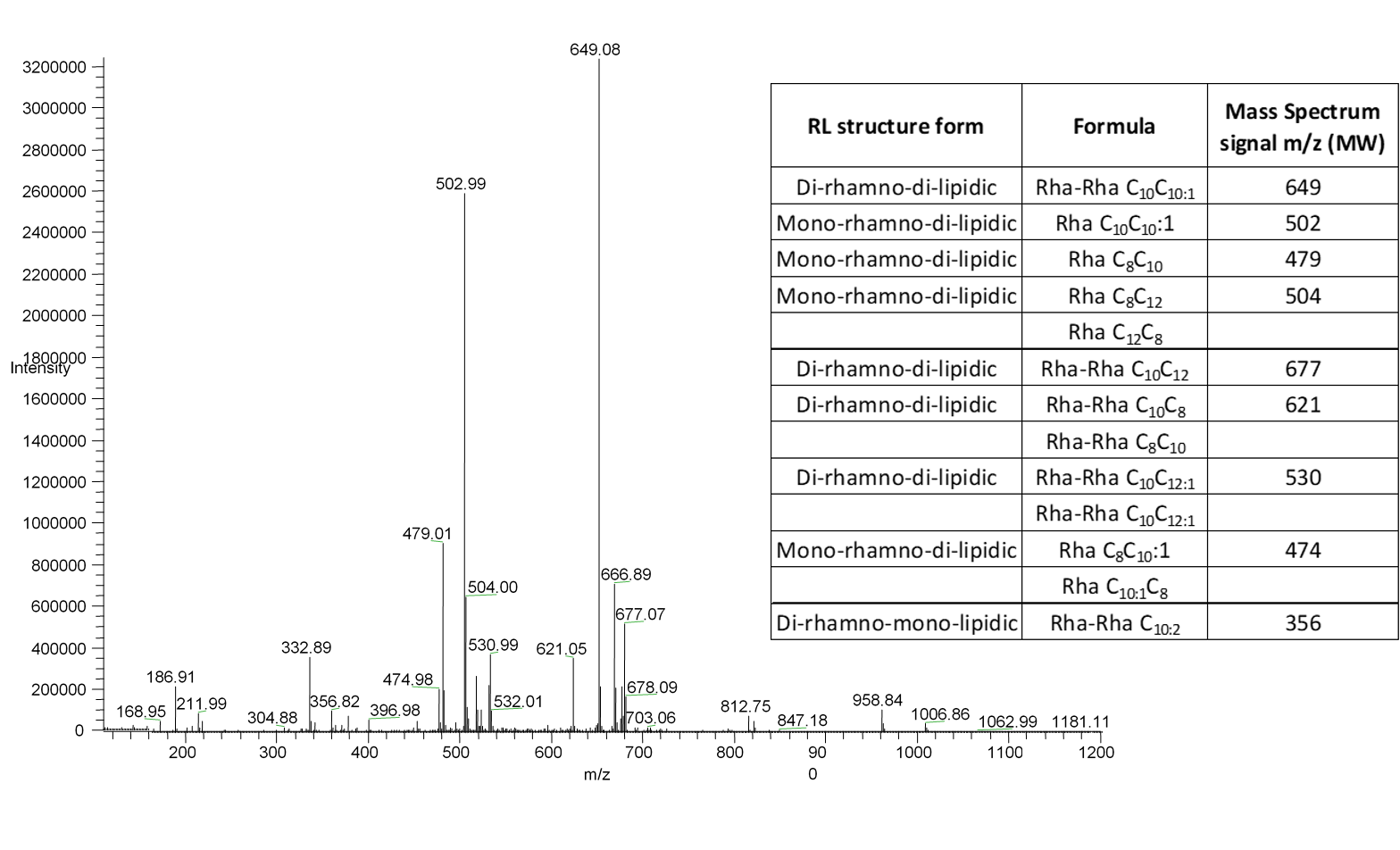

Supplement: FIGURE S1 — HPLC analysis spectrum of rhamnolipid and table of results with approximate probable molecular weights. [file Image_1.TIF]

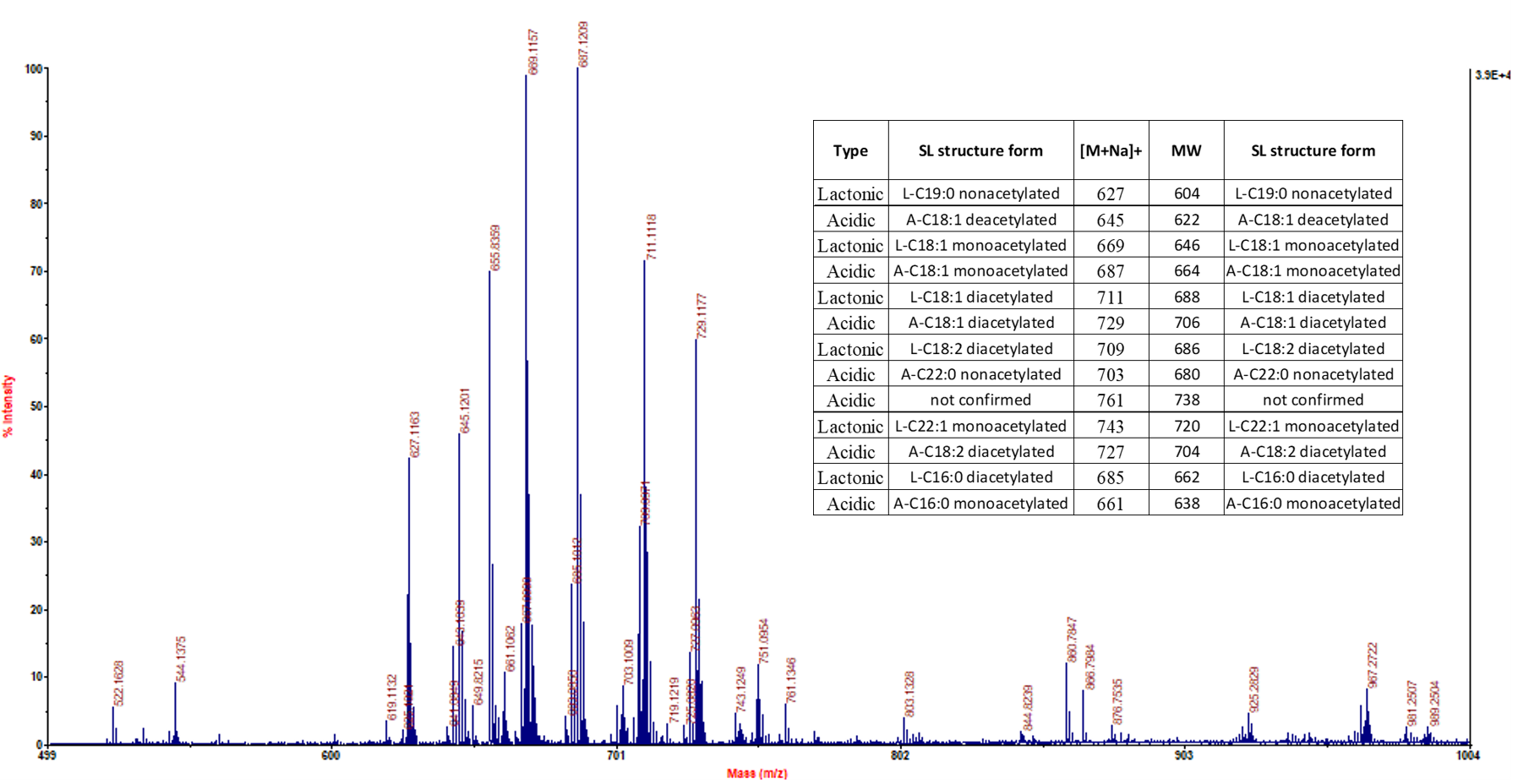

Supplement: FIGURE S2 — MALDI-TOF analysis spectrum of sophorolipid and table of results with approximate probable molecular weights. [file Image_2.TIF]

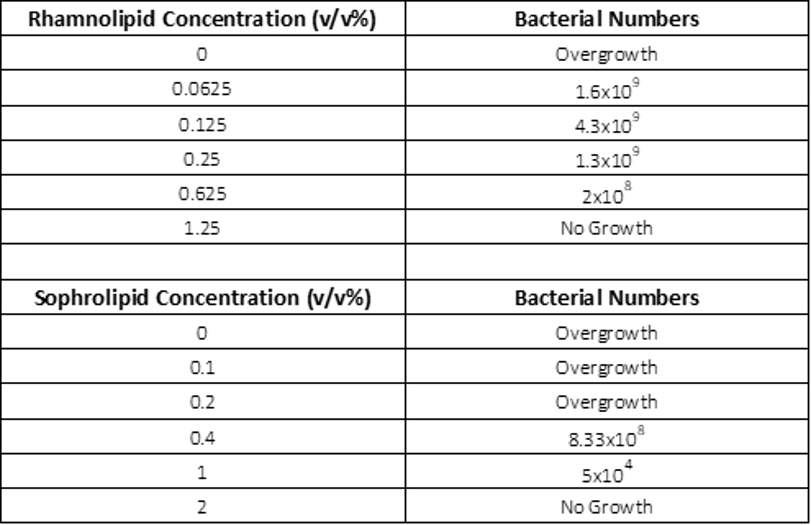

Supplement: FIGURE S3 — Results for microdilution assay: The bacterial number are in CFU/ml. [file Image_3.TIF]

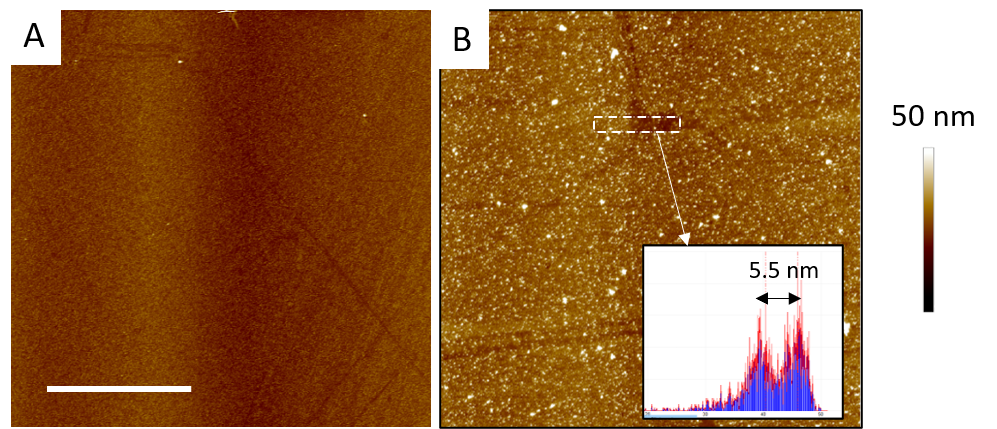

Supplement: FIGURE S4 — 30 μm TAFM height images of glass surface (A) and PEI-coated glass surface (B). The insert shows a height histogram originating from a sample region, dashed rectangle, around a scratch. [file Image_4.tif]
